# Self-Organized Men’s Mental Health and Well-Being Groups—An Emerging Social Development

**DOI:** 10.1177/15579883261426876

**Published:** 2026-03-31

**Authors:** Richard Gater, Jonathan Scourfield, Rhiannon Evans, Paul Willis

**Affiliations:** 1 Cardiff University, Cardiff, UK

**Keywords:** emotional openness, masculinities, mental health, peer support, well-being groups

## Abstract

Men’s mental health is a national and global concern amid consistently higher suicide rates and rising demand and long waiting times for state-funded services. Simultaneously, groups supporting men’s health and well-being, such as Men’s Sheds and community-led self-help and peer-support initiatives, have expanded. Despite their growth, research on the role of men’s peer-support groups in tackling mental health is still in the early stages, with limited understanding of how they function. To begin to address this gap, we surveyed men’s mental health and well-being groups throughout Wales (UK) in 2024, to describe their target audience, theoretical basis, activities, and delivery strategies. The online survey included fixed-response items and open-ended questions. Topics covered included group activities, participation frequency, types and numbers of participants, purposes and beliefs, structure and staffing, barriers, and publicity methods. Only 40% of groups reported offering practical activities while 79% reported promoting emotional openness, which could suggest a fairly direct approach to encouraging men to share personal thoughts and experiences is now more widespread than a “health by stealth” approach based around practical activities. Many groups reported rising participation despite operating without formally trained or salaried staff. Research is needed to understand how groups can be optimally delivered to achieve a balance between maintaining informal support structures and ensuring quality control. Further evidence is needed on a range of aspects, including group members’ experiences in these groups, program theory in practice, and the effectiveness of the groups in improving mental health.

## Background

Gender differences in mental health are complex. Boyd et al. (2015) report from research in Europe that internalizing disorders are more commonly found in women and externalizing disorders in men. In terms of suicidality, they found patterns to vary across countries. However, when it comes to death by suicide, the global rate in men is more than double that in women, and this pattern is found consistently across countries (World Health Organisation [WHO], 2025). Men’s mental health is therefore increasingly recognized in many countries as a health policy priority.

In responding to the need for mental health support, statutory health services in many countries are struggling to keep pace with increasing demand (e.g., British Medical Association, 2024). In the authors’ context of Wales, UK, Mind Cymru reports that over 2,000 people a month with moderate to severe mental health conditions are waiting more than 6 months for therapy (Mind Cymru, 2024a). The charity has called for greater availability of community and third-sector mental health services focused on prevention and peer support (Mind Cymru, 2024b). This is particularly relevant given the Welsh Government’s commitment to a society in which people’s physical and mental well-being is maximized (Welsh Government, 2021) and the emphasis on prevention in the Social Services and Well-being (Wales) Act 2014.

Despite growing recognition of men’s mental health challenges, many men remain reluctant to seek help, often denying psychological distress (Seidler et al., 2020). The reluctance of men to seek support for mental health problems is often attributed to adherence to traditional masculine norms, which privilege stoicism and self-reliance, promote emotional suppression, and can foster sexist ideology, resistance to gender equality, and homophobia (Connell, 2005). Instead, men have often been more inclined to engage in distracting and alternative coping strategies, such as substance use (Courtenay, 2011). With growing public awareness of gender’s influence on mental health, many countries have experienced a contemporary cultural examination of masculinity and challenges to men’s health improvement, and contemporary voices have called for men to adopt progressive forms of masculinity that emphasize emotional expressiveness and gender-egalitarian views (McQueen, 2017). Coincidingly, some recent academic research has highlighted a shift in men’s ideas of manhood in some contexts and an acceptance of softer expressions of masculinity, such as emotional openness, sensitivity, and the admission of vulnerability (Anderson, 2009; Gater, 2024; McCormack, 2014).

These challenges to men’s health are mirrored in the international growth of practice initiatives targeting men’s health and well-being, with formalized groups often seeking to support men in being more expressive. One of these is Men’s Sheds (Kelly et al., 2021), which now operate across twelve countries (AMSA, 2025). The approach originated in Australia in the 1990s in response to increasing concerns about men’s health. They are often led by staff members who have experience in health or social care. Men’s Sheds consist of a workshop environment in community settings that provide opportunities for hands-on activities such as woodwork and metalworking, as well as social activities, including playing cards or watching films (Barbagallo et al., 2023).

Through this range of traditional male activities, Men’s Sheds are considered to create “health by stealth” (McGeechan et al., 2017), meaning that by engaging in activities together, men are encouraged to communicate with one another. The traditionally masculine nature of the activity is meant to allow them to be more open with each other in discussion (Milligan et al., 2016). However, Mackenzie et al. (2017) caution that Men’s Sheds’ environments and activities can also reinforce traditional masculine norms. Foettinger et al.’s (2022) mixed-methods systematic review on the effectiveness of Men’s Sheds in targeting self-rated health, social isolation, and well-being indicated that older men could benefit from Shed participation in connection with their mental health, well-being, and social isolation.

In recent years, there also appears to have been a rapid expansion in men’s mental health and well-being community-led informal self-help and peer-support groups, which operate outside formalized health and social care services and center on discussing emotional states and well-being, rather than this being a side-product as in a health-by-stealth approach. We are aware of these new groups anecdotally and through systematic web searching. Many appear to operate without any external funding and entail voluntary group structures established for mutual support to achieve specific aims (Katz & Bender, 1976). Examples from the UK include MenWalkTalk, which currently has 21 groups (MenWalkTalk, 2025) and Andy’s Man Club which operates in 240 locations (Andy’s Man Club, 2025). These community-led peer-support groups share commonalities with formal Men’s Sheds-type groups, including an emphasis on support from others with shared lived experience (Bellamy et al., 2017) and informal support structures that are reported to be more acceptable to men than professional interventions, as they create a safe and trusting environment and are perceived as more “male-friendly” (Robinson et al., 2015; Seidler et al., 2020). However, determining the full scope of these men’s mental health and well-being groups is challenging due to their community-driven, grassroots nature, focus on localized promotion, variations in the group titles and, in many cases, lack of affiliation with established bodies.

The idea of social interventions specifically for men has been around for a few decades, since the more widespread public awareness of second wave feminism and responses to this in health and social care practice (Featherstone et al., 2007). The politics of masculinity are contested (Clatterbaugh, 1990), so a range of approaches would be expected. Messner (1997) argues that all men’s movements can be positioned in relation to the three different dimensions of the costs of masculinity, men’s privilege, and differences between men. It is evident that there are a minority of organizations supporting men that follow a men’s rights approach as a backlash against feminism (Jordan, 2019). It is not known whether such an approach can be found among informal peer-support and self-help groups for men’s mental health and well-being, although this might not be surprising in light of the wider cultural context, including the overt hostility to feminist understanding of gender equality in the online “manosphere” (Ging, 2019). There has previously been UK scoping of practice with fathers, and with men who perpetrate domestic abuse (Scourfield et al., 2014; Scourfield & Dobash, 1999)—research that has included attention to gender ideology, as part of programs’ theoretical basis. However, we are not aware of any such studies of groups supporting men’s mental health and well-being.

Growing evidence supports self-help and peer-support interventions for mental health. Systematic reviews show that group peer support modestly aids recovery and is cost-effective alongside professional treatment (Fuhr et al., 2014; Lyons et al., 2021; Smit et al., 2023). However, empirical research focused on peer support for mental health challenges in men specifically remains in an emerging phase (Sharp et al., 2024), with the evidence base mainly limited to only Men’s Sheds. There is also a paucity of understanding of the availability, design and effectiveness of a range of approaches. To address this evidence gap, we carried out a survey of all known men’s mental health and well-being groups in Wales, UK. The main research question was:

**Research Question 1**: What are the target audience, theoretical basis, activities, and delivery strategies of men’s mental health and well-being groups?

## Methods

An online survey was conducted with men’s well-being groups across Wales, with the aim of describing the provision across the nation. Wales is a relatively small nation (around 3 million population) within the UK, with a distinct national identity, language and some devolved government, including for health and social care.

### Stakeholder Engagement and Study Development

The authors’ attendance at a suicide and self-harm prevention discovery workshop “SIDE-BY-SIDE” in Cardiff in 2023 facilitated a working relationship with the facilitators of the event, including the organizations Men’s Sheds Cymru and Helpu Cymru, who exist to promote mental health support in Wales for men specifically, via a web directory and social media content. This partnership resulted in a joint effort to create the survey, with contributions from both organizations in its design and distribution.

### Survey Administration and Content

The online survey was created using the Qualtrics platform. It included a combination of fixed-response, pre-coded items and open questions with free-text boxes. The topics contained in the questionnaire were: general information (name, location, contact details); group activities; frequency of activity; type of participants and how many; purpose, approach, and beliefs; structure, staffing, and barriers; and publicity methods.

### Population, Sampling, and Sample Size

Men’s Sheds Cymru and Helpu Cymru promoted the survey on social media and shared it electronically among their members via email, which included approximately 64 affiliates of Helpu Cymru and over 70 members of Men’s Sheds Cymru, an unknown number of whom were also in the Helpu Cymru list. In addition to this, one of the authors (Gater) had identified some additional Welsh groups from systematic internet searches. We estimate that the survey was sent to around 100 different organizations. It was also shared on the LinkedIn social media platform, which could have reached some others.

### Distribution and Completion

Data were collected between October and December 2024. Responses were received from 30 groups, two of which did not complete some sections of the survey. This gave an estimated response rate of 30%, based on comparing completed questionnaires with a systematic internet search for groups and intelligence from the two partner organizations. Seventeen out of the 22 Welsh local authority districts were mentioned by name, with five responses stating they covered multiple districts or broader regions such as “South, mid and West” Wales.

### Data Cleaning and Analysis

In a few instances, two responses were received from a single group. The first inclusion criterion was to select the fuller of the two responses, but if the level of detail in the two was similar, the most recent was selected, on the assumption that it might have been revised on reflection. Two responses were excluded because they came from services that only offered one-to-one professional support, as that was not the focus on the survey. The descriptive analysis produced frequencies only, in light of the small achieved sample. Data were not normally distributed, so for numerical data, medians were reported as summary statistics rather than means.

### Ethics

Cardiff University’s School of Social Sciences Research Ethics Committee provided ethical approval for the study. Participants were informed that the first section of the survey, which gathered general information about the groups and their activities, would be made public via the Helpu Cymru website, whereas the second section, which addressed purpose, approach, beliefs, structure, and staffing barriers, would only be accessible to the research team

## Results

This section outlines the survey findings, organized around four key themes: the groups’ target audience, theoretical basis, activities delivered, and delivery strategy.

### Target Audience

Groups were asked about the age group of men targeted by their service. Twenty-two groups (73%) said they were targeted to, and supportive of, men of all ages. One group said they were more geared toward younger men (aged under 40), one said middle-aged men (aged 40–60), and two said older men (aged over 60). A further two said they targeted a combination of both younger and middle-aged men, and two said both middle-aged and older men.

Regarding specific needs, 22 (73%) groups said they did not target any particular groups of men. Seven said they were open to all. Of those who did target specific groups, the most common responses were men who are very isolated (*n* = 7, 23%) and men with mental distress (*n* = 6, 20%). Less common responses were: fathers (*n* = 3); two said gay and bisexual men; two said homeless men; and one group reported being targeted on each of the following: disabled men, transgender men, victims of domestic abuse, and those seeking recovery from addiction.

In terms of attendance, groups were asked roughly what percentage of men attended from different age bands. For men under 40, the median was 20%; for men aged 40–60, the median was 27%; and for men aged over 60, the median was 10%. They were asked roughly how many men attended sessions, and the answers ranged from 4 to 40, with median number being 13.

Groups were also asked whether their membership had increased over the last 12 months. Twenty-three out of 28 responding (82%) said yes it had. Four (14%) said no it had not, and one did not know.

### Theoretical Basis

Groups reported on the theoretical basis of their service, considering the purpose, approach, and beliefs underlying their provision (Table 1).

**Table 1.** Purpose and Approach, and Beliefs the Group Is Based on.

Statements in questionnaire	Strongly agree	Agree	Neither agree nor disagree	Disagree	Strongly disagree	Total
**Purpose and approach**						
Our group exists to provide company for men	18 (64%)	5 (18%)	4 (14%)	0 (0%)	1 (4%)	28
It’s fine to drop in and out of attending our group	15 (56%)	4 (15%)	2 (7%)	2 (7%)	4 (15%)	27
We expect men to commit to regular attendance	4 (14%)	4 (14%)	6 (21%)	6 (21%)	8 (29%)	28
In our group we encourage men to open up about problems	11 (39%)	11 (39%)	4 (14%)	2 (7%)	0 (0%)	28
We set “ground rules” for our group such as not talking over others and keeping things confidential	9 (32%)	12 (43%)	5 (18%)	2 (7%)	0 (0%)	28
**Beliefs the group is based on**						
The behavior of many men causes problems for women and children	2 (7%)	10 (36%)	10 (36%)	5 (18%)	1 (4%)	28
Men need to reclaim their essential masculinity (i.e. what men truly are)	3 (11%)	2 (7%)	10 (36%)	9 (32%)	4 (15%)	28
The differences between groups of men (e.g. “race”/ethnicity and social class) are more significant than their common experiences	0 (0%)	2 (7%)	11 (40%)	8 (29%)	7 (25%)	28
Transgender men are men and should be included in men’s groups	9 (32%)	7 (25%)	8 (29%)	2 (7%)	2 (7%)	28
Our organization is supportive of women’s needs and rights	19 (66%)	8 (28%)	2 (7%)	0 (0%)	0 (0%)	29

In terms of purpose, 82% of the groups agreed (64% strongly) they provide companionship for men, and 79% agreed (39% strongly) their group encourages men to discuss their problems. There was no consensus on whether or not men would be expected to commit to regular attendance, but a small majority (56%) thought it would be fine for men to drop in and out of attending the group.

Responses on underpinning beliefs suggest a mixture of ideological positions among the groups. These include a minority who appear to hold views consistent with essentialist conceptions of masculinity that have shaped certain men’s movements since the 1980s. There is also no clear consensus among respondents that men’s behavior causes problems for women and children, with some respondents disagreeing, so being less sympathetic than other groups to feminism. Although there is not a clear pro-feminist consensus, almost all agreed with the last (uncontroversial) statement about support for women’s needs and rights, so could be assumed to not be strongly anti-feminist either.

Views were fairly supportive of trans men’s inclusion in men’s groups, although 14% did not agree with this and 29% were equivocal. Few groups were focused on differences between men, perhaps reflecting a lack of ethnic and sexual diversity in the sample. Reflecting the salience of culture wars, one respondent wrote in a free-text box that the survey was “overly woke.” Another wrote that “Some of our members do have misogynistic tendencies and can be disingenuous to ethnicity and transgender.”

### Group Activities and Frequency

Groups reported an array of activities, delivered with varying frequency, including walking, one-to-one support, sport, group discussion focused on sport, and practical activities. Overall, 40% of groups offered practical activities (Table 2), partly explained by the fact that nine of the 30 groups (30%) had “shed” in their title so were likely aligned with the Men’s Sheds network, which has a strong emphasis on this approach. Many of these also incorporated discussion as a means of facilitating conversations around personal issues. A focus on personal issues was seen in the 63% offering support groups and 30% offering one-to-one support.

**Table 2.** Group Activities^a^.

Activity	*n* (%)
Walking group	8 (27%)
Support group	19 (63%)
One-to-one support (including peer mentoring)	9 (30%)
Sport	2 (7%)
A discussion group mainly focused on talking about sport	1 (3%)
Some kind of practical activity (e.g. building, making things, gardening)	12 (40%)
Other (please add information in the box below)	17 (57%)
Total	30

a Respondents could select multiple options.

The second most popular option in response to the question regarding the activities provided by the groups was the “other” category. Most of these activities were in fact already captured by the main categories offered to respondents, but some respondents wanted to give more specific information. The free-text responses that were not as clearly covered by the main questionnaire categories were: domestic abuse recovery (three groups, two of which specified provision for men as victims); an educational focus, such as talks, lectures, workshops and training; a specialist focus on addiction; and additional creative activities such making podcasts and communal singing.

Also, although only three groups selected either sport-related category, the “other” category included a further five who offered physical activity which respondents had not classified as “sport”—fitness classes, yoga, cold water dipping, and e-bike cycling. So, in total, seven groups (23%) offered a physical activity, including sport.

Sixteen groups (53%) met once or twice a week, six (20%) once or twice a month, three (10%) offered daily activities, three (10%) offered structured programs of a set number of sessions, and four (13%) offered support flexibly, when needed. Some of these responses overlapped, where an organization offered more than one form of support. Table 3 contains the results on the format of group meetings, whether indoors, outdoors, online, or hybrid. Indoor meeting appears to be the dominant format, although half of the groups sometimes meet outdoors.

**Table 3.** Where Does the Group Meet?.

Questionnaire options	Some of the time	Most of the time	All of the time
	*n* (row %)		
In person indoors	8 (27%)	9 (30%)	9 (30%)
In person outdoors	15 (50%)	6 (20%)	4 (13%)
Full online (e.g. zoom)	8 (27%)	5 (17%)	0 (0%)
Some people in-person and some online (hybrid)	8 (27%)	3 (10%)	0 (0%)

### Delivery Strategy

Groups were asked about their delivery strategy, including organizational structure and staffing (Table 4). Relating to organizational structure, approximately nine out of ten did identified as non-profit, almost a third were a registered charity and a third were classified as a community interest company. Only a quarter reported receiving any public funding.

**Table 4.** Organizational Structure and Staffing.

Questionnaire item	Yes	No	Don’t know	Total
Does your organization aim to make a profit?	3 (11%)	25 (89%)	0 (0%)	28
Is your group closely linked to a coaching, self-help, or other well-being/mental health business?	9 (33%)	18 (67%)	0 (0%)	27
Is your organization a registered charity?	10 (36%)	17 (61%)	1 (4%)	28
Is your organization a community interest company?	11 (39%)	14 (50%)	3 (11%)	28
Does your organization receive any funding from a public body or a charity?	7 (25%)	19 (68%)	2 (7%)	28
Does your organization have any paid staff locally?	8 (29%)	20 (71%)	0 (0%)	28
Does your organization have any paid staff nationally?	3 (11%)	24 (86%)	1 (4%)	28
Does your organization rely on volunteers to run?	20 (71%)	8 (29%)	0 (0%)	28

In regard to staffing, a large majority of groups (over 70%) reported having no paid staff, either at the local or national level. When indicating the professional qualifications of their staff, only six (21%) said they did have such staff, with four of these being counselors. The rest either said no or left this box blank, which we take to mean they had no professionally qualified staff. This question was a free-text response, so it is not clear if the prevalence of qualifications is higher but has just not been reported.

Groups were asked “What would you say is the single biggest barrier to providing your group currently?” Funding was the most frequently mentioned issue, some also connecting this with the availability of a suitable, affordable venue. Time was another frequently cited factor, with some respondents noting groups were reliant on volunteer leaders, who might also be employed elsewhere. One wrote about a lack of people taking responsibility for tasks involved in running the group. Some others answered the question about barriers with reference to group members’ qualities, for example, there is a “mind-set of men—believing they don’t need support or that there’s no benefit unless they are struggling mentally,” and reference to stigma and “fear of not being believed.” In one group, there was the issue of some members having dementia, and although an inclusive approach was taken, this could have the unintended consequence of the group working “to the lowest level” which “could be frustrating for some other members.”

For publicizing the group, out of 28 responses, 25 used Facebook, 13 Instagram, 6 X (formerly Twitter), and 6 selected “other” social media (naming TikTok, Bluesky, LinkedIn, and WhatsApp). Eight groups had used articles in local press, 16 were listed on another organization’s website, 15 had their own website, 22 distributed flyers, and 8 selected “other,” with responses including word of mouth and having stalls at events.

## Discussion

This article has shared results from an online survey of men’s mental health and well-being groups throughout Wales, which aimed to describe the national provision. While the evidence is somewhat constrained by the small survey size and relatively low response rate, the findings expand existing knowledge on the activities, operational frameworks, structures, and beliefs of men’s well-being groups.

Nine of the 30 groups had “shed” in their title, so were presumed to be part of the Men’s Sheds network. Forty percent of the groups reported employing approaches to men’s well-being typically associated with these organizations, including using practical activities or a strategy referred to as “health by stealth” (McGeechan et al., 2017). This method encourages men to engage in shared activities that are deemed to be of interest to men, fostering communication and openness among them, which helps break down traditional masculine barriers to discussing health problems (Milligan et al., 2016; Robertson & Baker, 2017). However, contrary to the conventional perceptions that men are reluctant to discuss health issues (Robertson & Baker, 2017), talking about personal issues features strongly in the survey responses, as represented by support groups, one-to-one support, and the large percentage agreeing that men are encouraged to open up about problems. The willingness to engage in discussions about health represents a significant social development that warrants attention, particularly given its contradiction with traditional masculine norms and the associated suppression of emotion (Connell, 2005). This shift may have implications for mainstream health service provision for men, especially considering the increasing waiting times and high thresholds for accessing mainstream professionally-led mental health services.

Further deviation from emotionally illiterate and rigid conceptions of manhood is evident in the purpose, approach, and beliefs-related findings. These reveal no clear picture of groups being more intensely therapeutic, considering the range of views on committed attendance. Furthermore, from the group representatives’ survey responses, it is not clear that the groups are either strongly pro-feminist nor anti-feminist. However, many of the groups promote emotional openness, which aligns with softer expressions of masculinity that are perhaps influenced by feminist movements such as #MeToo and their cultural examination of masculinity (Brown, 2022), emphasizing the need for softer expressions of manhood. In terms of Messner’s (1997) terrain of the politics of masculinities, the group’s overall may be more focused on the costs of masculinity than on men’s privileges or the differences between men. The lack of focus on difference could potentially have an impact on the inclusion of socially marginalized groups.

Overall, these findings contribute to the evidence that is beginning to challenge the notions of “men don’t seek help” or “men don’t talk” about health problems. They lend tentative support to the notion that, under the right circumstances, men are able to discuss health concerns (Gough et al., 2021) and to research indicating softer expressions of masculinity and an admission of vulnerability among contemporary men (Anderson, 2009; Gater, 2024). These results indicate a need to revise the current picture of men’s mental health and well-being groups in the research literature, which has mainly centered on Men’s Sheds. Alternative provisions are emerging, diverse in structure and intent, that remain underexplored and insufficiently understood.

### Limitations

As noted earlier, the sample size (*n* = 30) is small, and the estimated response rate (30%) is relatively low. Declining response rates are unfortunately a global trend, explained at least in part by the proliferation of online surveys (Eggleston, 2024). The figures for group attendance and groups reporting an increase in numbers are based solely on self -report, which may have led to some exaggeration. Nevertheless, an increase seems credible in the face of prolonged wait times and tight eligibility for state-funded professional mental health services (Mind Cymru, 2024a), encouraging members to seek alternative resources. It should be noted that some questionnaire items are very open to interpretation.

### Implications

Mainstream services could potentially learn from these groups, which primarily operate on the basis of volunteers, and without employed professionals such as psychologists, counselors, and social workers. This provision could help address the crisis of demand for formal professional services outstripping supply. The lack of trained personnel might underscore the financial constraints faced by many of these organizations, however it may well be a conscious choice for informal support structures, as these are generally perceived to be more acceptable to men than professional interventions (Robinson et al., 2015; Seidler et al., 2020). There are also potential disadvantages of informal volunteer-led services, as functioning without qualified staff means limited regulation, and uncertainty concerning the quality, standardization, and consistency of service delivery (Sharp et al., 2024). Providing some support to these groups, such as modest funding—even a small amount could make a significant impact—may help them survive and mitigate the need for some costly acute health and social care services. Further assessment of these groups is important, to determine how to strike a balance that preserves informal support structures while ensuring quality control. Furthermore, the research adds to the emerging body of evidence suggesting that, when the appropriate conditions are present, men are both able and willing to engage in conversations about mental health.

## Conclusion

These largely self-organized groups of men, which seem in many cases to involve explicit emotional support, rather than only “health by stealth” through practical activities, are a new feature of grassroots, community-level mental health promotion. We need more research evidence on these groups, including well-funded studies with larger geographical coverage and better response rate than our own modest survey. Qualitative research is needed, including some independent observation to find out what these groups are actually doing, work on identifying program theory, and interviews with participants to document how they have experienced the groups and to ascertain the expectations on and responsibilities of volunteers. Further research is needed to explore what approaches are effective, for which individuals, under what circumstances, and the reasons behind their success. Quantitative evaluation of impact will be important, using comparative designs.
